# Osteocalcin-expressing endothelial progenitor cells and serum osteocalcin forms are independent biomarkers of coronary atherosclerotic disease severity in male and female patients

**DOI:** 10.1007/s40618-022-01744-3

**Published:** 2022-01-28

**Authors:** H. E. Shahrour, S. Al Fahom, G. Al-Massarani, A. R. AlSaadi, P. Magni

**Affiliations:** 1 Department of Biochemistry and Microbiology, Faculty of Pharmacy, Damascus University, Damascus, Syria; 2 Department Radiation Medicine, Pharmacological Studies Division, Atomic Energy Commission of Syria (AECS), Damascus, Syria; 3 Department of Internal Medicine, Cardiovascular Disease Section, Faculty of Medicine, Damascus University, Damascus, Syria; 4 Dipartimento di Scienze Farmacologiche e Biomolecolari, Università Degli Studi di Milano, Milan, Italy; 5 IRCCS MultiMedica, Sesto S. Giovanni, Milan, Italy; 6 DISFeB-UNIMI, via Balzaretti 9, 20133 Milan, Italy

**Keywords:** Coronary atherosclerotic disease, Endothelial progenitor cells, Osteocalcin, Undercarboxylated osteocalcin, Carboxylated osteocalcin, Cardiovascular risk factors

## Abstract

**Purpose:**

Osteocalcin (OC), an osteoblast-derived regulator of metabolic processes, and circulating early endothelial progenitor cells (EPC, CD34 − /CD133 + /KDR +) expressing OC (OC +) are potential candidates linking bone metabolism and the vasculature and might be involved in vascular atherosclerotic calcification. This study aimed at assessing the association of circulating levels of different OC forms and of EPCs count with disease severity in patients with documented coronary atherosclerosis (CAD).

**Methods:**

Patients (*n* = 59) undergoing coronary angiography were divided, according to stenosis severity, into (1) early coronary atherosclerosis (ECA) (*n* = 22), and (2) late coronary atherosclerosis (LCA) (*n* = 37). Total OC (TOC), carboxylated OC (cOC), undercarboxylated OC (unOC) were quantified by ELISA. EPC OC + count was assessed by flow cytometry.

**Results:**

EPC OC + counts showed significant differences between ECA and LCA groups. unOC and unOC/TOC ratio were inversely correlated with EPC OC + count. A significant decrease in TOC and unOC plasma levels was associated with higher cardiovascular risk factors (CVRFs) number. EPC OC + count was correlated with LDL-C, total cholesterol, and triglycerides, with a greater significance in the LCA group. No association between the different forms of circulating OC (TOC, ucOC, cOC) and severity of CAD was found.

**Conclusion:**

This study showed a significant association between EPCs (CD34 − /CD133 + /KDR + /OC +), CAD severity and CVRFs, suggesting an active role for EPC OC + in the development of CAD. An inverse correlation between TOC, ucOC, and number of CVRFs was observed, suggesting that OC, regardless of its carboxylation status, may be developed as a further cardiovascular risk biomarker.

**Supplementary Information:**

The online version contains supplementary material available at 10.1007/s40618-022-01744-3.

## Introduction

Atherosclerosis is associated with different risk factors, leads to vascular injury and, in conjunction with thrombosis, is the common cause of coronary artery disease (CAD). Although several studies have identified multiple risk factors, associated with the clinical manifestation of the disease [1, 2], such as elevated LDL cholesterol (LDL-C) levels, as well as pro-inflammatory components [3], it has not yet been proven that the successful management of these factors, alone or in combination, can completely eliminate or slow down the development of the disease, leading to the concept of cardiovascular (CV) residual risk. The knowledge and validation of additional novel risk factors and reliable predictive biomarkers to be added and integrated with those already established is thus urgently required to improve the accuracy of prevention and management of atherosclerosis-related cardiovascular disease (ASCVD).

Vascular calcification, a major manifestation of atherosclerosis, has long been assumed to result from passive precipitation of calcium and phosphate, but it is now considered to be an active cell-mediated process that results in organized extracellular matrix deposition by osteoblast-like cells [4]. Interestingly, there is an increasing evidence for the involvement of the skeleton in the pathophysiology of ASCVD [5]. The skeleton has indeed been recognized as an endocrine organ [6–9] involved in several metabolic processes, including maintaining normal levels of blood glucose and lipids [10, 11]. Recent studies identified an additional biological function of osteoblasts, that is focused on the actions of osteocalcin (OC) [12, 13]. OC is a vitamin K-dependent, osteoblast-derived protein, which exists in the circulation in two forms, carboxylated (cOC) and undercarboxylated (ucOC) [14–16]. Through the secretion of OC, the bone regulates glucose homeostasis [8, 13] and male reproductive functions [17, 18]. In addition, clinical studies suggest a more complex role for OC in human metabolism, which led to investigations exploring its associations with CV disease (CVD) [19–22], and to clarify whether OC is a vital mediator or a nonparticipant bystander in energy metabolism and vascular function [23–25]. Another candidate potentially providing a link between bone metabolism and CVD are endothelial progenitor cells (EPCs), which are nucleated bone marrow-derived cells, that can be mobilized in response to vascular injury, and contribute to vascular repair [26, 27]. Interestingly, OC expression by EPCs has an osteogenic potential and might be involved in the modulation of vascular atherosclerotic calcification [5]. Several studies, that examined OC-positive endothelial progenitor cells (EPCs) or performed histological staining for OC, reported that higher OC levels were associated with an increase of markers of atherosclerosis and calcification [28, 29], possibly indicating that these OC-positive EPCs are a promising biomarker for severity stratification of CAD [30].

Based on these findings, the present study was conducted to examine the relationship between the circulating level of the different OC forms and the count of circulating EPCs (CD34 − /CD133 + /KDR +) expressing OC (OC +) in a cohort of patients with documented coronary artery atherosclerosis of variable severity.

## Methods

### Study cohort

A cross-sectional study was conducted in a cohort of consecutive patients with coronary artery atherosclerosis. Patients were hospitalized at the cardiac catheterization department in Alassad University hospital (Damascus, Syria). Clinical and demographic data were used in accordance with hospital regulations and patient permission. The occurrence of hard physical work and/or sports practice were used as indicators of physical activity. Patients with impaired renal function, paralysis, debility, stay in bed for more than two weeks, previous hormonal or metabolic disorders, or taking medications known to influence bone or calcium metabolism, such as vitamin D, bisphosphonates, calcitonin, estrogen, corticosteroids, or warfarin, were excluded. This study was conducted in 59 patients (men 61%, women 39%) divided into two groups: (1) early coronary atherosclerosis (ECA), 22 patients (men 50%, women 50%), aged 55.2 ± 9.7 years, defined as patients with mild CAD (< 50% stenosis in any major epicardial arteries), and (2) late coronary atherosclerosis (LCA), 37 patients (men 68%, women 32%), aged 60.6 ± 10.4 years, defined as patients with severe, mono/multivessel CAD (> 50% stenosis in at least one or more major epicardial arteries).

Patient were grouped according to having one or more of the following cardiovascular risk factors (CVRFs): (1) history of hypertension: systolic blood pressure ≧140 mmHg, diastolic blood pressure ≧90 mmHg; (2) history of type 2 diabetes mellitus (T2DM): hemoglobin A1c ≧6.5%, fasting plasma glucose (FPG) ≧126 mg/dL; (3) history of hypercholesterolaemia: total cholesterol (TC) ≧200 mg/dL, LDL-C ≧100 mg/dL; (4) previous/current smoking; (5) obesity, body mass index ≧30 kg/m^2^; and (6) family history of premature CAD: CAD in first-degree relatives < 55 years (male) or < 65 years (female). Concomitant medications are summarized in Supplemental Table 1. The study was approved by the Human Research Ethics Committee, Faculty of Pharmacy, Damascus University (HRECPHARMDU); all participating subjects signed an informed consent.

### Measurement of circulating biomarkers and serum OC forms

In each blood sample, TC, HDL-C, triglyceride (TG) and FPG were measured according to standard automated clinical procedure. LDL-C was calculated according to the Friedewald formula. The serum levels of different OC forms in peripheral blood specimen were measured using ELISA testing. Specifically, we measured the serum concentration of ucOC (Glu-OC MK118, Takara Bio Europe SAS, France), cOC (Gla-OC MK111, Takara Bio Europe SAS, France), and TOC (hOST-ELISA KAP1381, DIAsource ImmunoAssays SA, Belgium). Since ucOC levels are affected by bone formation, the ucOC/TOC ratio was calculated to adjust for this effect. Normal range reference values for OC forms, obtained from 26 healthy subjects (38.6 ± 7.7 years), were: cOC: 7.29 ± 3.27 mg/mL; ucOC: 6.65 ± 1.78 mg/mL.

### Evaluation of circulating EPCs OC + 

Flow cytometry was used to count circulating EPCs that were CD34 − CD133 + KDR + OC + . Three mL of peripheral blood was withdrawn into vacuum tubes containing EDTA as anticoagulant. Blood samples (200 µL) were stained with mouse anti-human monoclonal antibodies conjugated with fluorochromes: Anti-OC-APC (R&D Systems), Anti-CD34-PerCP-Cy5.5 (BD Biosciences), Anti-CD133-PE (Miltenyi Biotec), and Anti-KDR-FITC (Miltenyi Biotec). Erythrocytes were then lysed and leukocytes were washed with PBS and resuspended in 950 µL PBS. After the addition of 50 µL of counting beads, the samples were analyzed using a flow cytometer (MACSQuant®, Miltenyi Biotec GmbH). Autofluorescence and isotype controls were also included. MACSQuant software was used to acquire and analyze flow cytometric data. A total of 100,000 events in the mononuclear gate were acquired. Counting beads were used to calculate the absolute count of EPCs that were CD34 − CD133 + KDR + OC + , after applying the appropriate gating.

### Statistical analysis

Data was analyzed using the Statistical Package for the Social Sciences v22 (SPSS, Inc., Chicago, Illinois). The Kolmogorov–Smirnov test was performed to assess the sample cumulative distribution. Quantitative continuous variables are presented as mean ± SD or median and interquartile range. Normally distributed variables were analyzed using the two independent samples *t*-test. To analyze non-normally distributed data, the Mann–Whitney *U* test was performed. Kruskal–Wallis test was used to compare the mean ranks of TOC, ucOC, and cOC in relation to the number of cardiovascular risk factors (CVRFs). Spearman correlation coefficient was used to assess the strength of the association among EPC, OC, and CVRFs. Statistical significance was set at *p* < 0.05.

## Results

### Characteristics of the study cohort

The demographic, clinical, and biochemical characteristics of the study subjects are shown in Table 1. Patients with LCA were older than ECA subjects (60.57 ± 10.42 vs. 55.23 ± 9.65 years, *p* = 0.013). The M/F ratio was 50:50% in the ECA group, but males were prevalent (68%) in the LCA group. LCA and ECA groups did not differ according to incidence of smoking, TC, HDL-C, LDL-C and TG. Patients with T2DM were significantly more in the LCA group than in the ECA group (*p* = 0.01). There were no significant differences in BMI, smoking, non-T2DM prevalence, FPG and lipid profile between the two groups.

**Table 1:** Demographic, anthropometric, and biochemical parameters of the study population

	ECA (*n* = 22)	LCA (*n* = 37)	*p* value
Age (yr)	55.23 ± 9.65	60.57 ± 10.42	**0.013**
Gender (M/F)	11/11	25/12	0.19
Sedentary lifestyle *n* (%)	16(72.7)	30(81.1)	0.45
Smoking *n* (%)	7(31.8)	11(29.7)	0.87
BMI (kg/m^2^)	30.45 ± 4.85	29.32 ± 4.43	0.44
SBP (mmHg)	128.6 ± 14.9	126.5 ± 7.5	0.61
DBP (mmHg)	70.5 ± 12.1	68.9 ± 9.9	0.70
TC (mg/dL)	156.50 ± 41.24	149.46 ± 44.99	0.51
HDL-C (mg/dL)	31.91 ± 8.86	27.95 ± 5.89	0.11
LDL-C (mg/dL)	88.45 ± 25.46	83.43 ± 28.44	0.71
TG (mg/dL)	159.36 ± 78.91	146.59 ± 85.36	0.17
FPG (mg/dL)	160.46 ± 91.08	166.81 ± 84.11	0.48
Non-T2DM *n* (%)	13(59.1)	14(37.8)	0.85
T2DM *n* (%)	9(40.9)	23(62.2)	**0.01**

Data are expressed as mean ± standard deviation. Significant *p* values are in bold

*ECA* early cardiovascular atherosclerosis; *LCA* late cardiovascular atherosclerosis; *M/F* male/female; *BMI* body mass index; *SBP* systolic blood pressure; *DBP* diastolic blood pressure; *TC* total cholesterol; *HDL-C* cholesterol bound to high density lipoproteins; *LDL-C* cholesterol bound to low-density lipoproteins; *TG* triglyceride; *FPG* fasting plasma glucose; *T2DM* type 2 diabetes mellitus

### Serum OC forms and circulating EPC OC + in coronary atherosclerosis

**Fig. 1:**
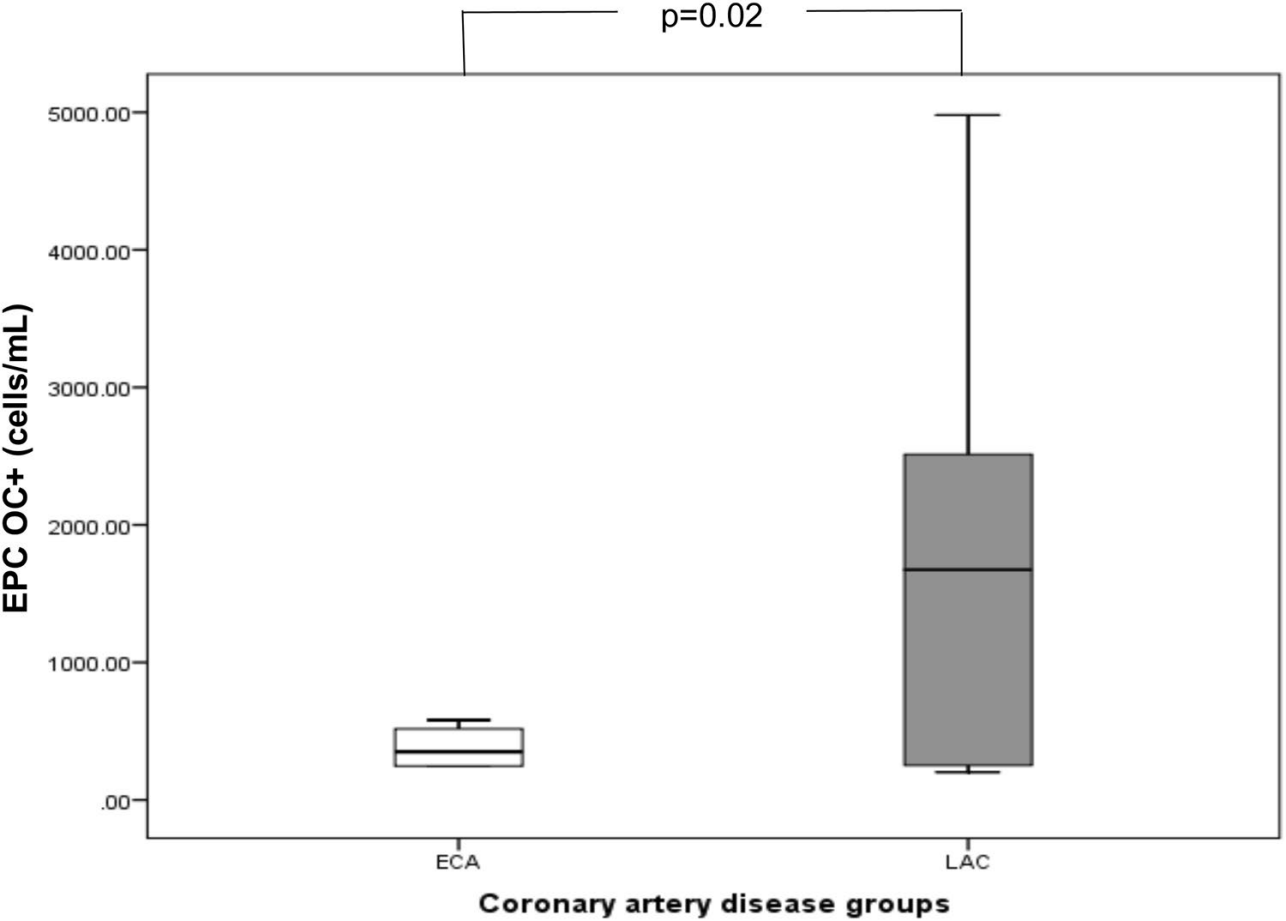
EPC OC + counts (median and range) in ECA and LCA groups, referred to patients above the 75th percentile of EPC OC + count (ECA: early cardiovascular atherosclerosis. LCA: late cardiovascular atherosclerosis. EPC OC + : endothelial progenitor cells expressing osteocalcin)

The circulating levels of OC forms (cOC, ucOC, TOC and ucOC/TOC) in the ECA group did not differ from those observed in the LCA group (Table 2). Regarding EPC OC + count, we observed that patients with EPC OC + counts above the 75th percentile showed significant differences in circulating EPC OC + counts between groups (*p* = 0.02), with LAC subjects showing more elevated EPC OC + counts (Table 2, Fig. 1).

**Table 2:** Circulating levels of osteocalcin forms and EPCs OC + count according to study groups

	ECA (*n* = 22)	LCA (*n* = 37)	*p* value
EPC OC + * (cells/mL)	359 (247–517)	1673 (251–2513)	0.02**
cOC (ng/mL)	7.35 ± 3.25	6.83 ± 2.85	0.60
ucOC (ng/mL)	2.09 ± 1.74	2.57 ± 1.86	0.27
ucOC/TOC	0.23 ± 0.12	0.29 ± 0.21	0.55
TOC (ng/mL)	9.44 ± 5.25	9.87 ± 6.72	0.99

Data are expressed as mean ± standard deviation or median and interquartile range

Significant *p* values are in bold. *defined as > 75th percentile, ** *t*-test

*ECA* early cardiovascular atherosclerosis; *LCA* late cardiovascular atherosclerosis; *EPC OC* + endothelial progenitor cells expressing osteocalcin; *cOC* carboxylated osteocalcin; *ucOC* undercarboxylated osteocalcin; *TOC* total osteocalcin

**Fig. 2:**
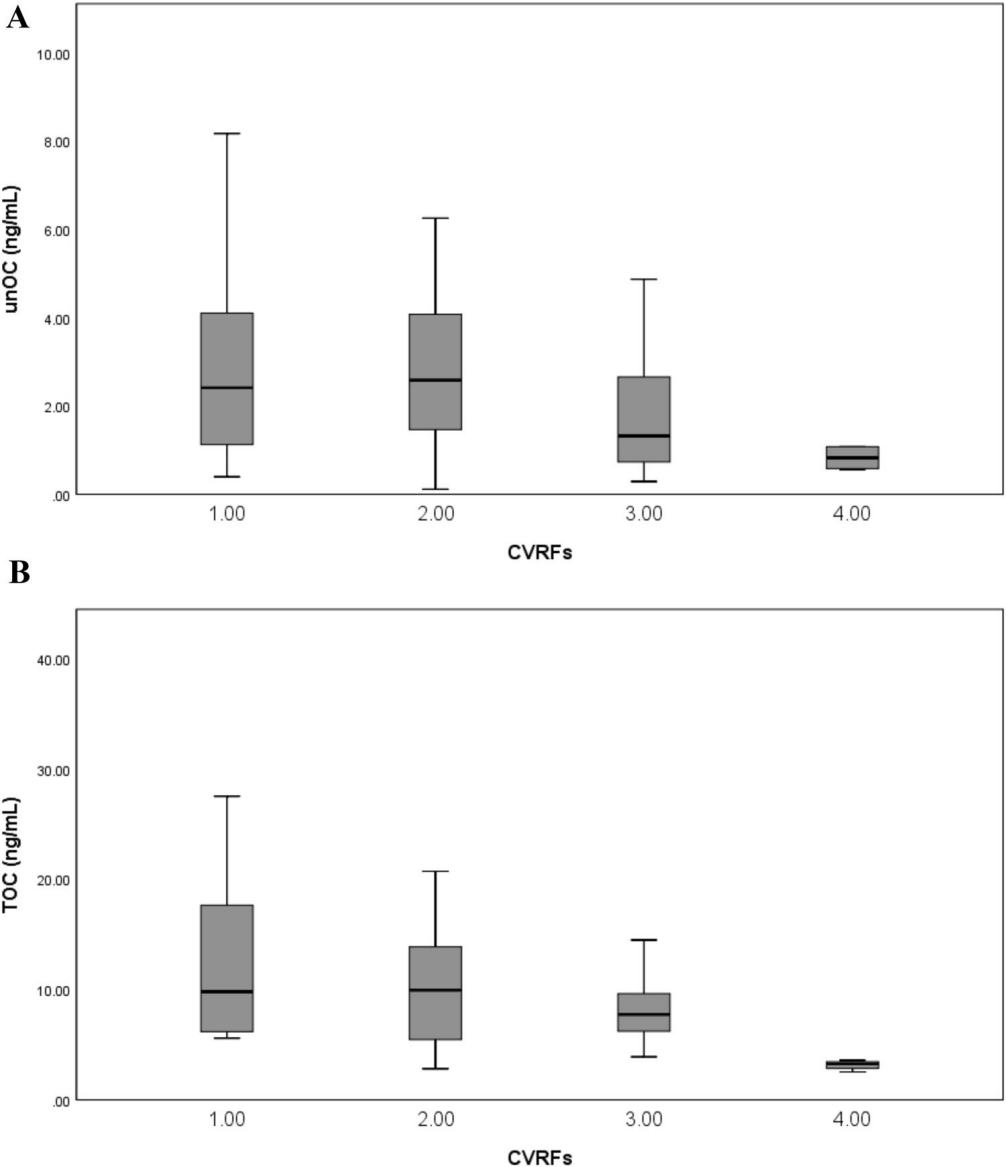
Relationship of **A** undercarboxylated osteocalcin (unOC) and **B** total osteocalcin (TOC) levels with the number of cardiovascular risk factors (CVRFs). unOC and TOC are expressed as mean ± SD. unOC and TOC levels significantly decreased in relationship with increasing number of CVRFs (*p* = 0.02 and *p* = 0.04, respectively; Kruskal–Wallis test)

**Fig. 3:**
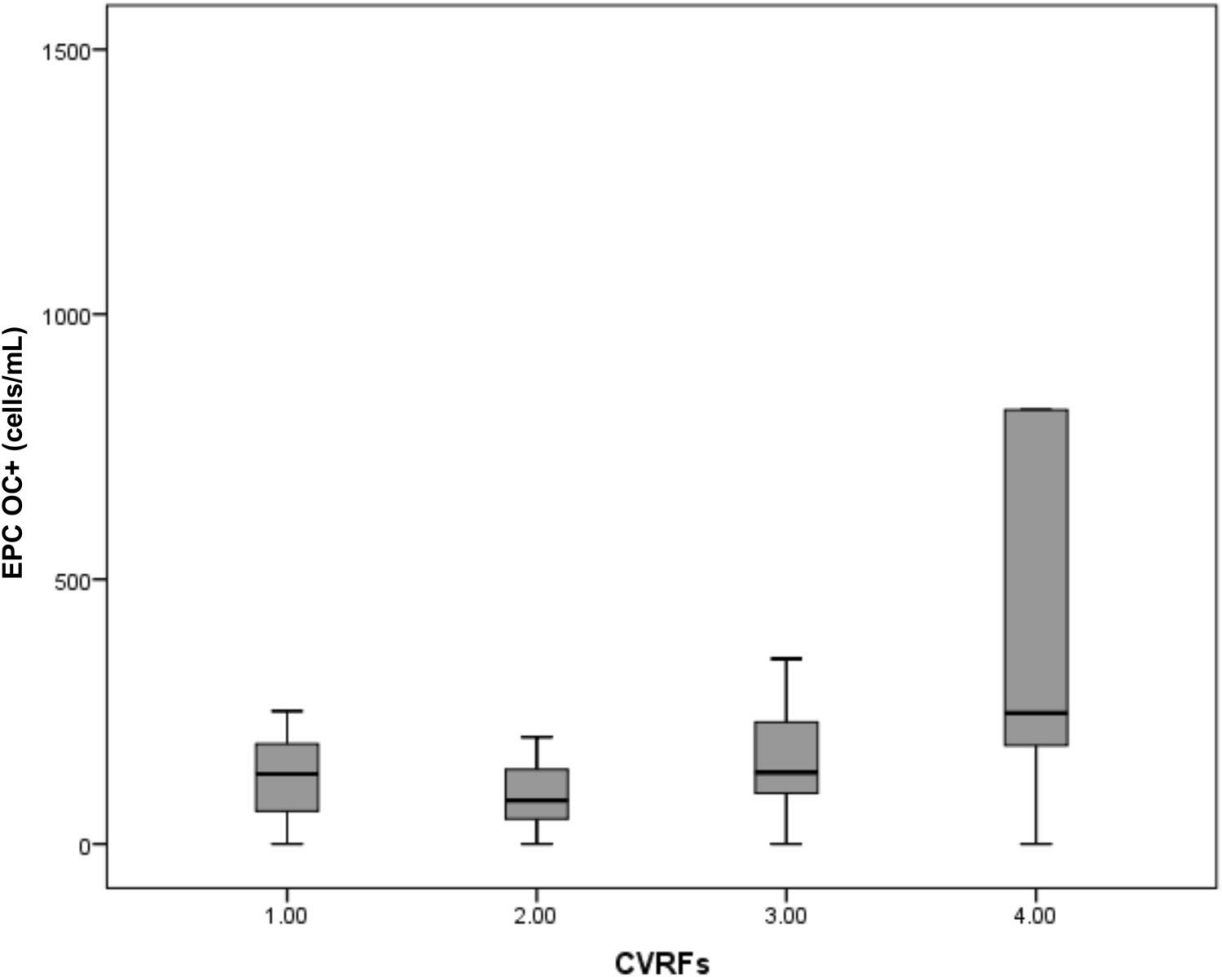
EPC OC + counts in relation to the number of cardiovascular risk factors (CVRFs). A significant increase in EPC OC + counts were observed with a higher number of CVRFs (*p* = 0.04, Kruskal–Wallis test). Data are expressed as mean ± SD

All patients had at least one CVRF. CVRFs number was associated with changes of unOC and TOC levels and EPC OC + count. A significant decrease of unOC levels was observed in parallel with the increase of number of CVRFs (*p* = 0.02) (Fig. 2A). A similar trend was also observed for TOC (*p* = 0.04) (Fig. 2B). On the contrary, a significant increase of EPC OC + count was observed with the increase of CVRFs number (*p* = 0.04) (Fig. 3).

### Correlation analysis of EPC OC + count with clinical parameters and levels of OC forms

In the whole study population, EPC OC + count was positively correlated with TC, LDL-C and TG, but not with other demographic and clinical parameters related to CVD risk (Table 3). In the LCA group, but not in the ECA group, the correlation of EPC OC + count with TC, LDL-C and TG was also present (Table 3). The correlation analysis between EPC OC + count and different OC forms is reported in Table 4. In the whole study population, EPC OC + count was inversely correlated with unOC levels and unOC/TOC (both *p* < 0.01). This correlation was also present in the LCA group for both unOC and unOC/TOC (*p* < 0.01).

**Table 3:** Correlation between EPC OC + and individual cardiovascular risk factors

	EPC OC +		
	Whole cohort (*n* = 59)	ECA (*n* = 22)	LCA (*n* = 37)
Age	0.005	− 0.166	0.021
BMI	0.001	0.043	0.000
Smoking	0.139	0.147	0.117
CVRFs	0.191	0.185	0.183
Gender	− 0.083	0.101	− 0.160
TC	0.459**	0.202	0.611**
HDL-C	0.120	0.213	0.069
LDL-C	0.408**	− 0.080	0.621**
TG	0.346**	0.339	0.373*
FPG	0.025	− 0.281	0.263

*p < 0.05; ** *p* < 0.01 (Spearman correlation coefficients)

*EPC OC* + endothelial progenitor cells expressing osteocalcin; *ECA* early cardiovascular atherosclerosis; *LCA* late cardiovascular atherosclerosis; *BMI* body mass index; *CVRFs* cardiovascular risk factors; *TC* total cholesterol; *HDL-C* cholesterol bound to high density lipoproteins; *LDL-C* cholesterol bound to low-density lipoproteins; *TG* triglyceride; *FPG* fasting plasma glucose

**Table 4:** Correlation between EPC OC + and different forms of osteocalcin

	EPC OC +		
	Whole cohort (*n* = 59)	ECA (*n* = 22)	LCA (*n* = 37)
cOC	0.138	0.246	0.036
ucOC	− 0.390**	− 0.199	− 0.505**
ucOC/TOC	− 0.426**	− 0.312	− 0.470**
TOC	− 0.004	0.142	− 0.088

**p < 0.01 (Spearman correlation coefficients)

*EPC OC* + endothelial progenitor cells expressing osteocalcin; *ECA* early cardiovascular atherosclerosis; *LCA* late cardiovascular atherosclerosis; *cOC* carboxylated osteocalcin; *ucOC* undercarboxylated osteocalcin; *TOC* total osteocalcin

## Discussion

It has recently been hypothesized that EPC OC + are involved in the mechanism of calcification by promoting abnormal vascular repair [19]. Moreover, circulating EPC OC + found in the peripheral blood have been shown to be able to activate calcification in vitro and in vivo [29]. Based on these assumptions, this study was designed to evaluate, for the first time to the best of our knowledge, all different forms of OC as well as EPCs expressing OC + counts in the context of patients with coronary atherosclerosis. Our study showed significant differences in circulating EPC OC + counts between LAC and ECA groups, with EPC OC + count increased with the severity of CAD, possibly suggesting an active role for EPC OC + in the development of atherosclerosis. This is consistent with several studies [28–33] demonstrating a significant positive correlation between OC-positive cells and increased vascular calcification or atherosclerosis.

In our study, an inverse correlation between ucOC, ucOC/TOC and EPC OC + in CAD was found. This indicates a potentially protective role for ucOC that is consistent with previous studies [34, 35], showing that circulating ucOC is, for example, inversely correlated with insulin resistance.

In the current study, we observed a new relationship between EPC OC + and lipid indexes, since EPC OC + positively correlated with TC, LDL-C, and TG, suggesting that EPC OC + count may play a role in the evaluation of the CV risk in patients with CAD. In a population-based study, EPC counts were positively correlated with CVRFs [36], and Ivaska et al. demonstrated an inverse correlation of these cells with CVRFs [37]. However, recent research has provided conflicting results [29].

There is little evidence describing the relationship between serum levels of ucOC, EPC OC + counts, and CVRFs [20]. In our study, we found TOC and ucOC to be similarly inversely correlated with increasing number of CVRFs. This suggests that OC, but not its carboxylation status, may be relevant to improve the metabolic profile and to reduce CV risk. This finding is supported by meta-analyses reporting that TOC and unOC are similarly and negatively correlated with FPG, and glycated hemoglobin (HbA1c) in humans [11]. We found that EPC OC + counts correlated with increasing number of CVRFs, a finding that supports the active role of EPC OC + in developing atherosclerosis.

We found no association between various forms of circulating OC (total, ucOC, or cOC) and severity of CAD, possibly due to the small sample size, which in this case represent a limitation of the study. A limitation of this study, intrinsic to the fact that it has been conducted in humans, is that the highlighted relationships could not be further explored in their possible causal association. This may be the object of future translational experiments. Next studies will also address the potential sex differences of such vascular calcification process, in the context of the well-known peculiarities in ASCVD risk and treatment [38]. In addition, since proprotein convertase subtilisin kexin type 9, in addition to control LDL-C levels [39], has been shown to contribute to vascular and valvular calcification [40], it will be interesting to further unveil its potential relationship with EPC OC + and OC.

## Conclusions

The present study measured the three independent forms of circulating OC and EPC OC + count in CAD patients. We found a significant association between circulating early EPCs (CD34 − CD133 + KDR + OC +), CAD severity, and CVRFs. This suggests an active role for EPC OC + in developing atherosclerosis. We showed a novel inverse correlation between ucOC and EPC OC + in CAD, indicating a protecting role for unOC. We also found an inverse correlation between TOC and ucOC and number of CVRFs, suggesting that OC, but not its carboxylation status, may be developed as a further cardiovascular risk biomarker. These findings have potential implications for the mechanisms of vascular calcification and for the development of novel markers to identify patient at low or high coronary atherosclerosis risk and suggest a novel link between bone metabolism and progression of coronary atherosclerosis.

## Supplementary Information

Below is the link to the electronic supplementary material.

Supplementary file1 (PDF 38 KB)
