# Supplementary material for: Osteocalcin-expressing endothelial progenitor cells and serum osteocalcin forms are independent biomarkers of coronary atherosclerotic disease severity in male and female patients
Source: J Endocrinol Invest. 2022 Jan 28;45(6):1173–80. doi: 10.1007/s40618-022-01744-3 (PMC9098612; doi:10.1007/s40618-022-01744-3)
Supplement: Supplementary file 1 — Supplementary file1 (PDF 38 KB) [file 40618_2022_1744_MOESM1_ESM.pdf]

Supplementary data

Supplementary Table 1. Concomitant medications

Medication	Patients (%)
Calcium antagonists	26
Beta Blockers	60
ACE-I	19
ARB	16
Nitrates	21
Diuretics	12
Hypoglycemic Agents	16
Statins	65
Anti-Platelets	63
Aspirin	65
Anticoagulants	5
(3-KAT) inhibitor	21
Other drugs	35

ACE-I: angiotensin-converting enzyme inhibitor
ARB: Angiotensin receptor blockers
3-KAT: 3-ketoacyl coenzyme A thiolase
